# Supplementary material for: Intra-Familial Phenotypic Heterogeneity and Telomere Abnormality in von Hippel- Lindau Disease: Implications for Personalized Surveillance Plan and Pathogenesis of VHL-Associated Tumors
Source: Front Genet. 2019 Apr 24;10:358. doi: 10.3389/fgene.2019.00358 (PMC6491623; doi:10.3389/fgene.2019.00358)
Supplement: Supplementary file 1 [file Table_1.DOCX]

**Table S1 Clinical and genetic characteristics of VHL patients involved in this study**

| **Family** | **Patient** | **Parents-children pairs** | **Off-spring pairs** | **Patient ID** | **Sex^a^** | **Birth year** | **Origin^b^** | **Mutation** | **Phenotype** | | | | | | **Age adjusted RTL** | **follow up** | **Survival status^c^** | **Cause of death** |
| --- | --- | --- | --- | --- | --- | --- | --- | --- | --- | --- | --- | --- | --- | --- | --- | --- | --- | --- |
|  |  |  |  |  |  |  |  |  | **Onset age** | **CHB** | **RA** | **RCC** | **PCT** | **PHEO** |  |  |  |  |
| 1 | Ⅰ-1 | 3 | 1 | 2 | 2 | 1944 | un | c.269A>T p.Asn90Ile | 33 | 33 | － | － | － | － | － | 34 | 1 | CHB |
|  | Ⅰ-2 |  |  | 5 | 2 | 1949 | un | c.269A>T p.Asn90Ile | 50 | － | － | － | 50 | － | 0.400 | 67 | 0 |  |
|  | Ⅱ-1 |  |  | 1 | 1 | 1973 | m | c.269A>T p.Asn90Ile | 28 | 28 | － | 38 | 38 | 38 | 0.397 | 43 | 0 |  |
|  | Ⅱ-2 |  |  | 6 | 1 | 1976 | m | c.269A>T p.Asn90Ile | － | － | － | － | － | － | 0.436 | 40 | 0 |  |
|  | Ⅲ-1 |  |  | 7 | 2 | 2003 | p | c.269A>T p.Asn90Ile | － | － | － | － | － | － | -0.226 | 13 | 0 |  |
| 2 | Ⅰ-1 | 1 | 0 | 36 | 2 | 1978 | n | c.256C>T p.Pro86Ser | 12 | － | 12 | 33 | 33 | － | 0.239 | 38 | 0 |  |
|  | Ⅱ-1 |  |  | 37 | 2 | 2003 | m | c.256C>T p.Pro86Ser | － | － | － | － | － | － | 0.472 | 13 | 0 |  |
| 3 | Ⅰ-1 | 3 | 4 | 46 | 1 | 1968 | un | c.349T>G p.Trp117Gly | 36 | 36 | 44 | 44 | 40 | － | 0.582 | 48 | 0 |  |
|  | Ⅰ-2 |  |  | 51 | 2 | 1960 | un | c.349T>G p.Trp118Gly | 46 | 47 | － | － | 46 | － | － | 47 | 1 | CHB |
|  | Ⅰ-3 |  |  | 53 | 1 | 1963 | un | c.349T>G p.Trp120Gly | 43 | 46 | 43 | 44 | 44 | － | － | 46 | 1 | RCC |
|  | Ⅰ-4 |  |  | 52 | 1 | 1949 | un | c.349T>G p.Trp119Gly | 47 | 47 | - | 61 | 59 | - | － | 62 | 1 | RCC |
|  | Ⅰ-5 |  |  | 50 | 2 | 1954 | un | c.349T>G p.Trp117Gly | 53 | － | － | - | 53 | - | 0.946 | 62 | 0 |  |
|  | Ⅱ-1 |  |  | 47 | 1 | 1996 | p | c.349T>G p.Trp117Gly | 11 | 12 | 11 | － | － | － | 0.486 | 20 | 0 |  |
|  | Ⅱ-2 |  |  | 49 | 2 | 1990 | m | c.349T>G p.Trp117Gly | － | － | － | － | － | － | 0.159 | 26 | 0 |  |
|  | Ⅱ-3 |  |  | 48 | 1 | 1995 | p | c.349T>G p.Trp117Gly | 13 | 13 | － | － | － | 18 | － | 21 | 0 |  |
| 4 | Ⅰ-1 | 4 | 3 | 55 | 2 | 1944 | un | c.292T>A p.Tyr98Asn | 50 | 50 | － | － | 62 | － | － | 62 | 1 | CHB |
|  | Ⅱ-1 |  |  | 54 | 1 | 1971 | m | c.292T>A p.Tyr98Asn | 40 | 40 | － | 40 | 40 | 40 | － | 45 | 0 |  |
|  | Ⅱ-2 |  |  | 56 | 2 | 1960 | m | c.292T>A p.Tyr98Asn | 51 | 51 | 51 | － | － | － | － | 56 | 0 |  |
|  | Ⅱ-3 |  |  | 57 | 2 | 1963 | m | c.292T>A p.Tyr98Asn | 40 | 40 | － | 43 | 43 | － | － | 53 | 0 |  |
|  | Ⅱ-4 |  |  | 58 | 1 | 1975 | m | c.292T>A p.Tyr98Asn | 16 | 27 | 16 | － | － | － | － | 27 | 1 | CHB |
| 5 | Ⅰ-1 | 1 | 0 | 103 | 1 | 1975 | n | c.257C>T p.Pro86Leu | 32 | 34 | 32 | 38 | 38 | － | － | 38 | 1 | RCC |
|  | Ⅱ-1 |  |  | 104 | 2 | 1996 | p | c.257C>T p.Pro86Leu | 18 | 18 | － | － | － | － | 0.174 | 20 | 0 |  |
| 6 | Ⅰ-1 | 1 | 0 | 111 | 1 | 1956 | p | c.194C>T p.Ser65Leu | 36 | 36 | － | － | － | － | － | 43 | 1 | CHB |
|  | Ⅱ-1 |  |  | 109 | 1 | 1982 | p | c.194C>T p.Ser65Leu | 21 | 21 | － | － | 30 | － | 0.675 | 34 | 0 |  |
| 7 | Ⅰ-1 | 2 | 1 | 115 | 2 | 1950 | un | c.269A>T p.Asn90Ile | 63 | 63 | － | 63 | 63 | － | － | 66 | 0 |  |
|  | Ⅱ-1 |  |  | 113 | 1 | 1982 | m | c.269A>T p.Asn90Ile | 20 | 20 | 30 | － | － | － | － | 34 | 0 |  |
|  | Ⅱ-2 |  |  | 114 | 1 | 1980 | m | c.269A>T p.Asn90Ile | 33 | 33 | － | － | － | － | － | 36 | 0 |  |
| 8 | Ⅰ-1 | 2 | 1 | 121 | 1 | 1957 | un | c.266T>C p.Leu89Pro | 40 | 40 | 40 | － | － | － | － | 56 | 1 | CHB |
|  | Ⅱ-1 |  |  | 120 | 1 | 1988 | p | c.266T>C p.Leu89Pro | 20 | 20 | － | － | 25 | － | － | 28 | 0 |  |
|  | Ⅱ-2 |  |  | 123 | 1 | 1973 | m | c.266T>C p.Leu89Pro | 38 | － | － | 38 | － | － | － | 43 | 0 |  |
|  | Ⅱ-3 |  |  | 125 | 1 | 1977 | m | c.266T>C p.Leu89Pro | 30 | 30 | － | － | － | － | － | 39 | 0 |  |
|  | Ⅲ-1 |  |  | 124 | 2 | 1991 | p | c.266T>C p.Leu89Pro | 16 | － | 16 | － | － | － | － | 25 | 0 |  |
| 9 | Ⅰ-1 | 3 | 1 | 141 | 2 | 1935 | un | c.194C>G p.Ser65Trp | 61 | 61 | － | － | － | － | － | 62 | 1 | CHB |
|  | Ⅱ-1 |  |  | 139 | 1 | 1965 | m | c.194C>G p.Ser65Trp | 48 | － | － | 48 | 48 | － | 0.441 | 51 | 0 |  |
|  | Ⅱ-2 |  |  | 142 | 1 | 1969 | m | c.194C>G p.Ser65Trp | 33 | 33 | － | － | － | － | － | 47 | 0 |  |
|  | Ⅲ-1 |  |  | 140 | 1 | 1992 | p | c.194C>G p.Ser65Trp | 22 | 22 | － | － | － | － | 0.063 | 24 | 0 |  |
| 10 | Ⅰ-1 | 3 | 1 | 151 | 1 | 1932 | un | c.245G>C p.Arg82Pro | 57 | 57 | － | － | － | － | － | 66 | 1 | CHB |
|  | Ⅱ-1 |  |  | 150 | 1 | 1973 | m | c.245G>C p.Arg82Pro | 26 | 40 | － | － | 26 | － | 0.280 | 43 | 0 |  |
|  | Ⅱ-2 |  |  | 152 | 1 | 1959 | m | c.245G>C p.Arg82Pro | 31 | 31 | － | － | － | － | － | 54 | 1 | CHB |
|  | Ⅲ-1 |  |  | 153 | 2 | 1985 | p | c.245G>C p.Arg82Pro | 21 | 21 | － | － | － | － | － | 31 | 0 |  |
| 11 | Ⅰ-1 | 2 | 1 | 164 | 2 | 1968 | un | c.293A>C p.Tyr98Ser | 45 | 45 | － | － | － | － | － | 45 | 1 | CHB |
|  | Ⅱ-1 |  |  | 162 | 2 | 1981 | m | c.293A>C p.Tyr98Ser | 27 | － | － | － | 27 | － | 0.264 | 35 | 0 |  |
|  | Ⅱ-2 |  |  | 163 | 2 | 1986 | m | c.293A>C p.Tyr98Ser | 28 | － | － | － | 28 | － | 0.986 | 30 | 0 |  |
| 12 | Ⅰ-1 | 3 | 2 | 193 | 2 | 1960 | m | c.263G>C p.Trp88Ser | 44 | 44 | － | － | － | － | － | 56 | 0 |  |
|  | Ⅱ-1 |  |  | 194 | 1 | 1987 | m | c.263G>C p.Trp88Ser | 26 | 26 | － | － | － | － | 0.142 | 29 | 0 |  |
|  | Ⅱ-2 |  |  | 188 | 1 | 1983 | p | c.263G>C p.Trp88Ser | 25 | 25 | 25 | 30 | 30 | － | 0.164 | 33 | 0 |  |
|  | Ⅱ-3 |  |  | 190 | 2 | 1981 | p | c.263G>C p.Trp88Ser | 24 | 24 | － | 31 | － | － | － | 35 | 0 |  |
|  | Ⅲ-1 |  |  | 290 | 1 | 2009 | p | c.263G>C p.Trp88Ser | － | － | － | － | － | － | 0.522 | 7 | 0 |  |
|  | Ⅲ-2 |  |  | 291 | 2 | 2006 | p | c.263G>C p.Trp88Ser | － | － | － | － | － | － | 0.295 | 10 | 0 |  |
| 13 | Ⅰ-1 | 6 | 5 | 227 | 2 | 1953 | un | c.194C>T p.Ser65Leu | 59 | 59 | － | － | － | － | 0.536 | 63 | 0 |  |
|  | Ⅰ-2 |  |  | 228 | 1 | 1950 | un | c.194C>T p.Ser65Leu | 45 | 45 | － | 59 | 65 | － | 0.570 | 66 | 0 |  |
|  | Ⅰ-3 |  |  | 230 | 1 | 1959 | un | c.194C>T p.Ser65Leu | 51 | 51 | － | 51 | 51 | － | － | 57 | 0 |  |
|  | Ⅰ-4 |  |  | 234 | 2 | 1962 | un | c.194C>T p.Ser65Leu | 53 | 53 | 53 | － | － | － | 0.413 | 54 | 0 |  |
|  | Ⅰ-5 |  |  | 235 | 1 | 1966 | un | c.194C>T p.Ser65Leu | 34 | － | 49 | 49 | － | － | 0.446 | 50 | 0 |  |
|  | Ⅱ-1 |  |  | 225 | 2 | 1974 | m | c.194C>T p.Ser65Leu | 36 | － | － | 36 | 37 | － | 0.343 | 42 | 0 |  |
|  | Ⅱ-2 |  |  | 229 | 1 | 1975 | p | c.194C>T p.Ser65Leu | 22 | 22 | － | － | － | － | － | 23 | 1 | CHB |
|  | Ⅱ-3 |  |  | 231 | 1 | 1983 | p | c.194C>T p.Ser65Leu | 22 | 22 | － | 30 | 31 | － | 0.665 | 33 | 0 |  |
|  | Ⅱ-4 |  |  | 233 | 1 | 1986 | p | c.194C>T p.Ser65Leu | － | － | － | － | － | － | 0.342 | 30 | 0 |  |
|  | Ⅲ-1 |  |  | 226 | 2 | 1999 | m | c.194C>T p.Ser65Leu | － | － | － | － | － | － | 0.575 | 17 | 0 |  |
|  | Ⅲ-2 |  |  | 232 | 2 | 2010 | p | c.194C>T p.Ser65Leu | － | － | － | － | － | － | -0.004 | 6 | 0 |  |
| 14 | Ⅰ-1 | 2 | 2 | 284 | 1 | 1955 | un | c.233A>G p.Asn78Ser | 55 | － | － | 55 | － | － | 0.643 | 61 | 0 |  |
|  | Ⅱ-1 |  |  | 282 | 1 | 1981 | p | c.233A>G p.Asn78Ser | 34 | 34 | － | 34 | 34 | － | 0.434 | 35 | 0 |  |
|  | Ⅱ-2 |  |  | 283 | 2 | 1988 | p | c.233A>G p.Asn78Ser | 27 | － | － | 27 | 27 | － | 0.767 | 28 | 0 |  |
|  | Ⅱ-3 |  |  | 285 | 1 | 1960 | un | c.233A>G p.Asn78Ser | 19 | 19 | － | － | － | － | － | 41 | 1 | CHB |
| 15 | Ⅰ-1 | 3 | 0 | 303 | 1 | 1957 | un | c.256C>T p.Pro86Ser | 54 | － | － | 54 | － | － | － | 59 | 0 |  |
|  | Ⅱ-1 |  |  | 342 | 2 | 1988 | p | c.256C>T p.Pro86Ser | － | － | － | － | － | － | 0.266 | 28 | 0 |  |
|  | Ⅱ-1 |  |  | 302 | 1 | 1983 | p | c.256C>T p.Pro86Ser | 30 | 31 | － | 30 | － | － | 0.374 | 33 | 0 |  |
|  | Ⅲ-1 |  |  | 304 | 2 | 2005 | p | c.256C>T p.Pro86Ser | 11 | － | 11 | － | － | － | 0.145 | 11 | 0 |  |
| 16 | Ⅰ-1 | 1 | 0 | 307 | 1 | 1954 | un | c.349T>A p.Trp117Arg | 26 | 26 | － | 47 | 59 | － | 0.675 | 62 | 0 |  |
|  | Ⅱ-1 |  |  | 306 | 2 | 1980 | p | c.349T>A p.Trp117Arg | 18 | 18 | － | － | 25 | － | 0.497 | 36 | 0 |  |
| 17 | Ⅰ-1 | 1 | 1 | 339 | 2 | 1966 | un | c.269A>T p.Asn90Ile | 39 | － | － | 39 | 39 | － | 0.871 | 50 | 0 |  |
|  | Ⅰ-2 |  |  | 340 | 1 | 1975 | un | c.269A>T p.Asn90Ile | 28 | 28 | － | － | － | － | － | 28 | 1 | CHB |
|  | Ⅱ-1 |  |  | 338 | 1 | 1990 | m | c.269A>T p.Asn90Ile | 16 | 16 | 23 | 26 |  |  | 0.772 | 26 | 0 |  |
| 18 | Ⅰ-1 | 1 | 0 | 387 | 1 | 1959 | p | c.239G>T pSer80Ile | 43 | 43 | － | － | － | － | － | 57 | 0 |  |
|  | Ⅱ-1 |  |  | 391 | 2 | 1986 | p | c.239G>T pSer80Ile | 29 | 29 | － | － | － | 29 | － | 30 | 0 |  |
| 19 | Ⅰ-1 | 4 | 3 | 9 | 1 | 1947 | un | c.499C>T p.Arg167Trp | 54 | 65 | － | 54 | 67 | － | 1.315 | 69 | 0 |  |
|  | Ⅱ-1 |  |  | 8 | 2 | 1973 | p | c.499C>T p.Arg167Trp | 37 | 37 | － | 37 | 37 | 39 | 0.563 | 43 | 0 |  |
|  | Ⅱ-2 |  |  | 10 | 2 | 1968 | p | c.499C>T p.Arg167Trp | 22 | 22 | － | － | － | － | － | 22 | 1 | CHB |
|  | Ⅱ-3 |  |  | 11 | 2 | 1974 | p | c.499C>T p.Arg167Trp | 36 | 39 | － | － | 36 | － | 1.288 | 42 | 0 |  |
|  | Ⅱ-4 |  |  | 12 | 2 | 1978 | p | c.499C>T p.Arg167Trp | 32 | － | 34 | － | 32 | － | － | 38 | 0 |  |
| 20 | Ⅰ-1 | 2 | 1 | 61 | 2 | 1962 | m | c.500G>A p.Arg167Gln | 29 | 29 | － | 46 | － | 46 | － | 54 | 0 |  |
|  | Ⅰ-2 |  |  | 63 | 2 | 1955 | m | c.500G>A p.Arg167Gln | 50 | 50 | － | － | － | － | － | 61 | 0 |  |
|  | Ⅱ-1 |  |  | 65 | 2 | 1984 | m | c.500G>A p.Arg167Gln | 14 | 24 | 14 | － | － | － | － | 32 | 0 |  |
|  | Ⅱ-2 |  |  | 64 | 1 | 1980 | m | c.500G>A p.Arg167Gln | 15 | 15 | － | 25 | － | － | － | 36 | 0 |  |
| 21 | Ⅰ-1 | 1 | 0 | 67 | 2 | 1959 | un | c.499C>T p.Arg167Trp | － | － | － | － | － | － | － | 57 | 0 |  |
|  | Ⅱ-1 |  |  | 66 | 1 | 1983 | m | c.499C>T p.Arg167Trp | 24 | － | － | 27 | 24 | 24 | 0.797 | 33 | 0 |  |
| 22 | Ⅰ-1 | 3 | 1 | 108 | 2 | 1930 | un | c.500G>A p.Arg167Gln | 54 | － | － | 54 | － | － | － | 54 | 1 | RCC |
|  | Ⅱ-1 |  |  | 105 | 1 | 1956 | m | c.500G>A p.Arg167Gln | 52 | 52 | － | － | － | － | 0.603 | 60 | 0 |  |
|  | Ⅱ-2 |  |  | 107 | 2 | 1958 | m | c.500G>A p.Arg167Gln | 14 | － | － | － | － | 14 | 0.595 | 58 | 0 |  |
|  | Ⅲ-1 |  |  | 106 | 1 | 1982 | p | c.500G>A p.Arg167Gln | 26 | － | 26 | － | － | 31 | 0.275 | 34 | 0 |  |
| 23 | Ⅰ-1 | 1 | 0 | 132 | 1 | 1962 | un | c.486C>G p.Cys162Trp | 40 | 40 | － | － | － | － | － | 54 | 0 |  |
|  | Ⅱ-1 |  |  | 131 | 2 | 1982 | p | c.486C>G p.Cys162Trp | 30 | 30 | － | － | － | － | 0.235 | 34 | 0 |  |
| 24 | Ⅰ-1 | 3 | 1 | 149 | 2 | 1948 | un | c.482G>A p.Arg161Gln | 56 | － | － | － | 56 | 56 | － | 68 | 0 |  |
|  | Ⅱ-1 |  |  | 146 | 2 | 1976 | m | c.482G>A p.Arg161Gln | 17 | 37 | － | 37 | 37 | 17 | 0.263 | 40 | 0 |  |
|  | Ⅱ-2 |  |  | 147 | 1 | 1980 | m | c.482G>A p.Arg161Gln | 8 | 27 | 26 | － | － | 8 | 0.193 | 36 | 0 |  |
|  | Ⅲ-1 |  |  | 148 | 2 | 2013 | p | c.482G>A p.Arg161Gln | － | － | － | － | － | － | -0.094 | 3 | 0 |  |
| 25 | Ⅰ-1 | 2 | 1 | 158 | 2 | 1953 | un | c.500G>A p.Arg167Gln | 35 | 35 | － | － | － | － | － | 63 | 0 |  |
|  | Ⅱ-1 |  |  | 156 | 2 | 1981 | m | c.500G>A p.Arg167Gln | 24 | 24 | － | 32 | 33 |  | 0.190 | 35 | 0 |  |
|  | Ⅱ-2 |  |  | 157 | 1 | 1988 | m | c.500G>A p.Arg167Gln | 20 | 20 | － | － | － | 26 | 0.093 | 28 | 0 |  |
| 26 | Ⅰ-1 | 1 | 0 | 166 | 2 | 1960 | un | c.499C>T p.Arg167Trp | 25 | 38 | 25 | － | 52 | － | 0.753 | 56 | 0 |  |
|  | Ⅱ-1 |  |  | 165 | 2 | 1984 | m | c.499C>T p.Arg167Trp | 13 | 26 | 13 | － | － | － | 0.164 | 32 | 0 |  |
| 27 | Ⅰ-1 | 3 | 2 | 170 | 2 | 1952 | p | c.486C>G p.Cys162Trp | 30 | 30 | 54 | 50 | － | － | － | 61 | 1 | RCC |
|  | Ⅰ-2 |  |  | 172 | 2 | 1961 | p | c.486C>G p.Cys162Trp | 37 | 45 | 53 | － | － | - | － | 55 | 0 |  |
|  | Ⅱ-1 |  |  | 169 | 2 | 1979 | m | c.486C>G p.Cys162Trp | 28 | 28 | － | 35 | 35 | － | 0.152 | 37 | 0 |  |
|  | Ⅱ-2 |  |  | 168 | 1 | 1981 | m | c.486C>G p.Cys162Trp | 33 | － | － | 33 | 33 | － | 0.184 | 35 | 0 |  |
|  | Ⅲ-1 |  |  | 171 | 2 | 2002 | m | c.486C>G p.Cys162Trp | 12 | 12 | － | － | － | － | － | 14 | 0 |  |
| 28 | Ⅰ-1 | 2 | 1 | 174 | 2 | 1953 | un | c.500G>A p.Arg167Gln | 45 | 45 | － | 49 | － | － | － | 59 | 1 | RCC |
|  | Ⅱ-1 |  |  | 173 | 1 | 1976 | m | c.500G>A p.Arg167Gln | 22 | － | － | 22 | － | 36 | － | 40 | 0 |  |
|  | Ⅱ-2 |  |  | 175 | 2 | 1981 | m | c.500G>A p.Arg167Gln | 27 | － | 30 | 27 | － | － | 0.043 | 35 | 0 |  |
| 29 | Ⅰ-1 | 1 | 0 | 200 | 2 | 1956 | m | c.499C>T p.Arg167Trp | 36 | － | － | － | － | 36 | 0.555 | 60 | 0 |  |
|  | Ⅱ-1 |  |  | 199 | 2 | 1986 | m | c.499C>T p.Arg167Trp | 16 | 16 | － | － | － | － | 0.175 | 30 | 0 |  |
| 30 | Ⅰ-1 | 2 | 0 | 221 | 1 | 1952 | un | c.509T>A p.Val170Asp | － | － | － | － | － | － | 0.559 | 64 | 0 |  |
|  | Ⅱ-1 |  |  | 219 | 2 | 1976 | p | c.509T>A p.Val170Asp | 23 | 23 | － | 39 | 39 | － | 1.279 | 40 | 0 |  |
|  | Ⅱ-2 |  |  | 220 | 2 | 1976 | p | c.509T>A p.Val170Asp | 36 | － | － | 36 | － | － | － | 36 | 1 | RCC |
| 31 | Ⅰ-1 | 2 | 2 | 266 | 1 | 1940 | un | c.499C>T p.Arg167Trp | 58 | － | － | － | － | 58 | － | 72 | 1 | PHEO |
|  | Ⅰ-2 |  |  | 267 | 1 | 1949 | un | c.499C>T p.Arg167Trp | 52 | － | － | － | - | 52 | － | 66 | 1 | PHEO |
|  | Ⅰ-3 |  |  | 270 | 1 | 1954 | un | c.499C>T p.Arg167Trp | - | － | － | － | － | - | 0.813 | 62 | 0 |  |
|  | Ⅱ-1 |  |  | 265 | 1 | 1966 | p | c.499C>T p.Arg167Trp | 26 | － | － | － | － | 26 | 0.822 | 50 | 0 |  |
|  | Ⅱ-2 |  |  | 268 | 1 | 1979 | p | c.499C>T p.Arg167Trp | 36 | － | － | － | － | 36 | 0.780 | 37 | 0 |  |
|  | Ⅲ-1 |  |  | 269 | 1 | 2012 | p | c.499C>T p.Arg167Trp | － | － | － | － | － | － | 1.193 | 4 | 0 |  |
| 32 | Ⅰ-1 | 1 | 0 | 344 | 2 | 1984 | un | c.499C>T p.Arg167Trp | 12 | 31 | － | 31 | 31 | 12 | 0.480 | 32 | 0 |  |
|  | Ⅱ-1 |  |  | 346 | 2 | 2012 | m | c.499C>T p.Arg167Trp | － | － | － | － | － | － | － | 4 | 0 |  |
| 33 | Ⅰ-1 | 1 | 2 | 13 | 1 | 1951 | m | c.533T>G p.Leu178Arg | 39 | 39 | 57 | 57 | 57 | － | － | 62 | 1 | CHB |
|  | Ⅰ-2 |  |  | 14 | 2 | 1949 | m | c.533T>G p.Leu178Arg | 40 | 40 | － | － | － | - | － | 67 | 0 |  |
|  | Ⅰ-3 |  |  | 15 | 1 | 1953 | m | c.533T>G p.Leu178Arg | 25 | - | 25 | 54 | － | - | － | 63 | 0 |  |
|  | Ⅱ-1 |  |  | 17 | 1 | 1979 | p | c.533T>G p.Leu178Arg | 30 | － | － | 30 | － | － | － | 37 | 0 |  |
| 34 | Ⅰ-1 | 1 | 0 | 129 | 1 | 1970 | m | c.452T>G p.Ile151Ser | 22 | 22 | － | 28 | － | － | 0.563 | 46 | 0 |  |
|  | Ⅱ-1 |  |  | 128 | 1 | 1996 | p | c.452T>G p.Ile151Ser | 14 | 14 | － | － | － | － | 0.307 | 20 | 0 |  |
| 35 | Ⅰ-1 | 2 | 1 | 137 | 2 | 1962 | un | c.388G>C p.Val130Leu | － | － | － | － | － | － | 0.241 | 54 | 0 |  |
|  | Ⅱ-1 |  |  | 135 | 1 | 1983 | m | c.388G>C p.Val130Leu | 30 | 30 | － | － | － | － | 0.170 | 33 | 0 |  |
|  | Ⅱ-2 |  |  | 136 | 1 | 1984 | m | c.388G>C p.Val130Leu | － | － | － | － | － | － | 0.186 | 32 | 0 |  |
| 36 | Ⅰ-1 | 1 | 0 | 71 | 2 | 1970 | n | deletion | 28 | 28 | 41 | 42 | 42 | 42 | 0.789 | 46 | 0 |  |
|  | Ⅱ-1 |  |  | 72 | 2 | 1997 | m | deletion | 16 | 16 | － | － | － | － | － | 19 | 0 |  |
| 37 | Ⅰ-1 | 2 | 1 | 75 | 2 | 1964 | m | deletion | 18 | 18 | － | 31 | 31 | － | 2.087 | 52 | 0 |  |
|  | Ⅰ-2 |  |  | 76 | 2 | 1968 | m | deletion | 46 | － | － | 46 | － | － | 1.110 | 46 | 1 | RCC |
|  | Ⅱ-1 |  |  | 77 | 2 | 1988 | m | deletion | － | － | － | － | － | － | 1.457 | 28 | 0 |  |
|  | Ⅱ-2 |  |  | 78 | 1 | 2008 | m | deletion | － | － | － | － | － | － | 1.312 | 8 | 0 |  |
| 38 | Ⅰ-1 | 1 | 0 | 84 | 2 | 1969 | p | deletion | 44 | － | － | 44 | 44 | － | － | 47 | 0 |  |
|  | Ⅱ-1 |  |  | 83 | 2 | 1987 | m | deletion | 26 | － | － | － | 26 | － | － | 29 | 0 |  |
| 39 | Ⅰ-1 | 1 | 0 | 92 | 1 | 1944 | un | deletion | 50 | 50 | － | － | — | － | － | 72 | 0 |  |
|  | Ⅱ-1 |  |  | 91 | 1 | 1975 | p | deletion | 31 | 37 | 31 | － | 37 | － | 0.355 | 41 | 0 |  |
| 40 | Ⅰ-1 | 1 | 0 | 93 | 2 | 1985 | un | deletion | 24 | 29 | 24 | － | 26 | － | 0.450 | 31 | 0 |  |
|  | Ⅱ-1 |  |  | 94 | 1 | 2013 | m | deletion | － | － | － | － | － | － | 0.266 | 3 | 0 |  |
| 41 | Ⅰ-1 | 1 | 0 | 185 | 1 | 1967 | un | deletion | 25 | 25 | 29 | － | － | － | － | 39 | 1 | CHB |
|  | Ⅱ-1 |  |  | 184 | 1 | 1991 | p | deletion | 15 | 16 | 15 | － | 22 | － | 0.035 | 25 | 0 |  |
| 42 | Ⅰ-1 | 3 | 1 | 208 | 2 | 1958 | p | deletion | 55 | 55 | － | － | － | － | 0.463 | 58 | 0 |  |
|  | Ⅱ-1 |  |  | 209 | 2 | 1984 | m | deletion | 24 | 24 | － | 31 | 31 | － | 0.198 | 32 | 0 |  |
|  | Ⅱ-2 |  |  | 210 | 2 | 1989 | m | deletion | 23 | 23 | － | － | 26 | － | 0.150 | 27 | 0 |  |
|  | Ⅲ-1 |  |  | 315 | 1 | 2011 | m | deletion | － | － | － | － | － | － | -0.113 | 5 | 0 |  |
| 43 | Ⅰ-1 | 1 | 0 | 237 | 1 | 1946 | un | deletion | 68 | － | － | 68 | － | － | － | 69 | 1 | RCC |
|  | Ⅱ-1 |  |  | 236 | 2 | 1972 | p | deletion | 33 | 33 | － | 40 | 40 | － | 0.361 | 44 | 0 |  |
| 44 | Ⅰ-1 | 1 | 1 | 239 | 2 | 1959 | un | deletion | 29 | 29 | － | － | － | － | － | 29 | 1 | CHB |
|  | Ⅰ-2 |  |  | 240 | 1 | 1963 | un | deletion | 28 | 28 | - | 50 | － | - | － | 53 | 0 |  |
|  | Ⅱ-1 |  |  | 238 | 2 | 1983 | m | deletion | 31 | 31 | － | 32 | 32 | － | 0.278 | 33 | 0 |  |
| 45 | Ⅰ-1 | 3 | 2 | 247 | 1 | 1960 | m | deletion | 48 | － | － | 48 | 48 | 48 | 0.432 | 55 | 1 | RCC |
|  | Ⅰ-2 |  |  | 249 | 1 | 1963 | m | deletion | 36 | 36 | 46 | － | － | － | － | 53 | 0 |  |
|  | Ⅰ-3 |  |  | 251 | 1 | 1968 | m | deletion | 20 | 30 | 20 | 41 | － | － | － | 41 | 1 | RCC |
|  | Ⅱ-1 |  |  | 248 | 1 | 1980 | p | deletion | 19 | 19 | 27 | － | － | － | － | 36 | 0 |  |
|  | Ⅱ-2 |  |  | 250 | 1 | 1995 | p | deletion | 16 | － | 16 | － | － | － | － | 21 | 0 |  |
|  | Ⅱ-3 |  |  | 252 | 1 | 1997 | p | deletion | 16 | － | 16 | － | － | － | － | 19 | 0 |  |
| 46 | Ⅰ-1 | 1 | 0 | 254 | 1 | 1958 | un | deletion | － | － | － | － | － | － | 0.594 | 58 | 0 |  |
|  | Ⅱ-1 |  |  | 253 | 2 | 1987 | p | deletion | 27 | 27 | － | 27 | 27 | － | 0.334 | 29 | 0 |  |
| 47 | Ⅰ-1 | 1 | 1 | 277 | 2 | 1957 | m | deletion | 35 | 35 | － | 43 | 43 | － | － | 59 | 0 |  |
|  | Ⅰ-2 |  |  | 278 | 2 | 1961 | m | deletion | 17 | 17 | 48 | － | - | 55 | － | 55 | 0 |  |
|  | Ⅱ-1 |  |  | 276 | 1 | 1982 | m | deletion | 16 | 16 | 24 | 22 | 22 | － | － | 34 | 0 |  |
| 48 | Ⅰ-1 | 3 | 1 | 298 | 1 | 1960 | un | deletion | － | － | － | － | － | － | 1.246 | 56 | 0 |  |
|  | Ⅱ-1 |  |  | 295 | 1 | 1990 | p | deletion | 14 | 25 | 14 | － | － | － | 0.248 | 26 | 0 |  |
|  | Ⅱ-2 |  |  | 296 | 2 | 1984 | p | deletion | 28 | － | － | 32 | 28 | － | 0.363 | 32 | 0 |  |
|  | Ⅲ-1 |  |  | 297 | 1 | 2011 | m | deletion | － | － | － | － | － | － | 0.242 | 5 | 0 |  |
| 49 | Ⅰ-1 | 2 | 0 | 313 | 1 | 1941 | un | deletion | 30 | 30 | － | － | － | － | － | 32 | 1 | CHB |
|  | Ⅱ-1 |  |  | 311 | 2 | 1965 | p | deletion | 49 | 51 | 49 | 51 | 51 | － | 0.547 | 51 | 0 |  |
|  | Ⅲ-1 |  |  | 312 | 2 | 1987 | m | deletion | 13 | 13 | － | － | － | － | － | 18 | 1 | CHB |
| 50 | Ⅰ-1 | 1 | 0 | 317 | 2 | 1941 | un | deletion | 42 | 42 | － | － | － | － | 0.793 | 75 | 0 |  |
|  | Ⅱ-1 |  |  | 316 | 1 | 1975 | m | deletion | 19 | 19 | － | － | 34 | － | 0.634 | 41 | 0 |  |
| 51 | Ⅰ-1 | 1 | 0 | 329 | 2 | 1968 | m | deletion | 35 | 35 | － | － | － | － | － | 41 | 1 | CHB |
|  | Ⅱ-1 |  |  | 328 | 1 | 1989 | m | deletion | 27 | － | － | 27 | 29 | － | 0.331 | 27 | 0 |  |
| 52 | Ⅰ-1 | 3 | 0 | 335 | 1 | 1963 | un | deletion | 38 | 41 | － | 38 | － | － | － | 41 | 1 | CHB |
|  | Ⅰ-2 |  |  | 336 | 1 | 1963 | un | deletion | 38 | 38 | 40 | 47 | 40 | － | － | 52 | 1 | CHB |
|  | Ⅱ-1 |  |  | 333 | 2 | 1987 | p | deletion | 23 | 23 | － | 28 | 25 |  | 0.350 | 29 | 0 |  |
|  | Ⅱ-2 |  |  | 337 | 2 | 1989 | p | deletion | 19 | 27 | 19 | 26 | － | － | － | 27 | 0 |  |
|  | Ⅲ-1 |  |  | 334 | 1 | 2015 | m | deletion | － | － | － | － | － | － | － | 1 | 0 |  |
| 53 | Ⅰ-1 | 3 | 3 | 377 | 1 | 1942 | m | deletion | 55 | － | － | 55 | 55 | － | － | 62 | 1 | RCC |
|  | Ⅰ-2 |  |  | 379 | 2 | 1964 | m | deletion | 45 | 45 | － | 47 | 47 | － | － | 47 | 1 | RCC |
|  | Ⅰ-3 |  |  | 378 | 1 | 1962 | m | deletion | 38 | 38 | 42 | 42 | － | - | － | 48 | 1 | RCC |
|  | Ⅱ-1 |  |  | 380 | 2 | 1974 | P | deletion | 36 | － | － | 36 | 36 | － | － | 42 | 0 |  |
|  | Ⅱ-2 |  |  | 381 | 1 | 1980 | P | deletion | 26 | 26 | － | － | － | － | － | 26 | 1 | CHB |
|  | Ⅱ-3 |  |  | 382 | 2 | 2000 | m | deletion | 12 | － | － | － | 12 | － | － | 16 | 0 |  |
| 54 | Ⅰ-1 | 1 | 0 | 134 | 2 | 1952 | un | c.329A deletion Framshift | 29 | 29 | 29 | － | － | － | － | 45 | 1 | CHB |
|  | Ⅱ-1 |  |  | 133 | 1 | 1981 | m | c.329A deletion Framshift | 18 | 18 | 18 | － | － | － | 0.780 | 35 | 0 |  |
| 55 | Ⅰ-1 | 1 | 0 | 155 | 2 | 1965 | un | c.436 insGG Framshift | 32 | 32 | － | 38 | － | 38 | － | 51 | 0 |  |
|  | Ⅱ-1 |  |  | 154 | 1 | 1989 | m | c.436 insGG Framshift | 21 | 25 | － | 23 | 21 | － | 0.123 | 27 | 0 |  |
| 56 | Ⅰ-1 | 4 | 4 | 176 | 1 | 1963 | un | c.239 delGTCCGCG Framshift | 32 | 32 | － | 32 | 52 | 50 | 0.516 | 53 | 0 |  |
|  | Ⅰ-2 |  |  | 180 | 1 | 1956 | un | c.239 delGTCCGCG Framshift | 52 | － | 52 | 52 | － | － | － | 57 | 1 | RCC |
|  | Ⅰ-3 |  |  | 179 | 2 | 1954 | un | c.239 delGTCCGCG Framshift | 22 | 22 | － | － | － | - | － | 22 | 1 | CHB |
|  | Ⅱ-1 |  |  | 177 | 2 | 1990 | p | c.239 delGTCCGCG Framshift | 17 | － | 17 | 21 | 18 | 19 | － | 26 | 0 |  |
|  | Ⅱ-2 |  |  | 178 | 1 | 1996 | p | c.239 delGTCCGCG Framshift | － | － | － | － | － | － | － | 20 | 0 |  |
|  | Ⅱ-3 |  |  | 181 | 2 | 1986 | p | c.239 delGTCCGCG Framshift | 22 | － | 22 | 22 | － | － | － | 30 | 0 |  |
|  | Ⅱ-4 |  |  | 182 | 2 | 1993 | p | c.239 delGTCCGCG Framshift | 18 | 19 | 18 | － | － | － | － | 23 | 0 |  |
| 57 | Ⅰ-1 | 2 | 1 | 197 | 1 | 1951 | un | c.205 insG Framshift | 58 | － | － | 58 | 58 | － | 0.657 | 65 | 0 |  |
|  | Ⅱ-1 |  |  | 195 | 2 | 1983 | p | c.205 insG Framshift | 31 | 31 | － | － | － | － | 0.204 | 33 | 0 |  |
|  | Ⅱ-2 |  |  | 196 | 1 | 1982 | p | c.205 insG Framshift | 32 | 32 | － | 32 | 32 | － | 0.401 | 34 | 0 |  |
| 58 | Ⅰ-1 | 3 | 2 | 214 | 1 | 1938 | un | c.433-437 delCAGCC Framshift | 25 | 25 | － | － | － | － | － | 60 | 1 | CHB |
|  | Ⅱ-1 |  |  | 211 | 1 | 1964 | p | c.433-437 delCAGCC Framshift | 26 | 26 | － | 45 | 45 | － | 0.430 | 52 | 0 |  |
|  | Ⅱ-2 |  |  | 212 | 1 | 1959 | p | c.433-437delCAGCC Framshift | 29 | 29 | － | 48 | 48 | － | － | 57 | 0 |  |
|  | Ⅱ-3 |  |  | 213 | 1 | 1962 | p | c.433-437delCAGCC Framshift | 27 | 27 | － | － | － | － | － | 53 | 1 | CHB |
| 59 | Ⅰ-1 | 2 | 0 | 243 | 1 | 1948 | un | c.224-6delTCT Framshift | 42 | 42 | － | － | － | － | － | 42 | 1 | CHB |
|  | Ⅱ-1 |  |  | 241 | 2 | 1977 | p | c.224-6delTCT Framshift | 26 | 28 | 26 | 37 | 28 | － | 0.298 | 39 | 0 |  |
|  | Ⅲ-1 |  |  | 242 | 1 | 1997 | m | c.224-6delTCT Framshift | － | － | － | － | － | － | 0.422 | 19 | 0 |  |
| 60 | Ⅰ-1 | 1 | 0 | 274 | 1 | 1985 | m | c.224-6delTCT Framshift | 21 | 21 | － | － | － | － | 0.547 | 30 | 1 | CHB |
|  | Ⅱ-1 |  |  | 275 | 1 | 2015 | p | c.224-6delTCT Framshift | － | － | － | － | － | － | 0.641 | 1 | 0 |  |
| 61 | Ⅰ-1 | 1 | 1 | 300 | 2 | 1979 | m | c.480delG Framshift | 22 | 22 | － | 27 | 27 |  | 0.470 | 37 | 0 |  |
|  | Ⅰ-2 |  |  | 299 | 1 | 1981 | m | c.480delG Framshift | 23 | - | 23 | 26 | 26 | - | 0.427 | 35 | 0 |  |
|  | Ⅱ-1 |  |  | 301 | 1 | 2011 | m | c.480delG Framshift | － | － | － | － | － | － | 0.236 | 5 | 0 |  |
| 62 | Ⅰ-1 | 1 | 1 | 320 | 2 | 1948 | un | c.224-6delTCT Framshift | 57 | 57 | － | － | － | － | － | 68 | 0 |  |
|  | Ⅱ-1 |  |  | 318 | 2 | 1978 | m | c.224-6delTCT Framshift | 28 | 38 | － | － | 28 | － | 0.664 | 38 | 0 |  |
|  | Ⅱ-2 |  |  | 319 | 2 | 1968 | un | c.224-6delTCT Framshift | 34 | 34 | － | － | － | - | － | 47 | 1 | CHB |
| 63 | Ⅰ-1 | 1 | 1 | 345 | 1 | 1953 | p | c.160delA Framshift | 50 | － | － | 50 | 50 | 50 | 0.893 | 63 | 0 |  |
|  | Ⅰ-2 |  |  | 347 | 1 | 1962 | p | c.160delA Framshift | 50 | － | - | 50 | - | 50 | － | 54 | 0 |  |
|  | Ⅱ-1 |  |  | 348 | 1 | 1979 | p | c.160delA Framshift | － | － | － | － | － | － | － | 37 | 0 |  |
| 64 | Ⅰ-1 | 2 | 3 | 360 | 2 | 1959 | u | c.224-6delTCT Framshift | 42 | 42 | － | 42 | － | － | － | 43 | 1 | RCC |
|  | Ⅰ-2 |  |  | 361 | 2 | 1945 | u | c.224-6delTCT Framshift | 54 | 54 | － | － | － | － | － | 55 | 1 | CHB |
|  | Ⅰ-3 |  |  | 364 | 2 | 1950 | u | c.224-6delTCT Framshift | 44 | 44 | － | － | － | - | － | 45 | 1 | CHB |
|  | Ⅰ-4 |  |  | 370 | 2 | 1954 | u | c.224-6delTCT Framshift | 24 | 24 | － | － | － | - | － | 25 | 1 | CHB |
|  | Ⅱ-1 |  |  | 359 | 1 | 1982 | m | c.224-6delTCT Framshift | 33 | 33 | － | 34 | 34 | － | － | 34 | 0 |  |
|  | Ⅱ-2 |  |  | 362 | 2 | 1969 | m | c.224-6delTCT Framshift | 33 | － | 33 | 33 | － | － | － | 47 | 0 |  |
| 65 | Ⅰ-1 | 2 | 1 | 395 | 1 | 1952 | u | c.224-6delTCT Framshift | 37 | 37 | － | 37 | 37 | － | － | 37 | 1 | CHB |
|  | Ⅱ-1 |  |  | 398 | 2 | 1999 | p | c.224-6delTCT Framshift | 16 | － | － | 16 | － | － | － | 17 | 0 |  |
|  | Ⅱ-2 |  |  | 399 | 1 | 2003 | p | c.224-6delTCT Framshift | 12 | 12 | － | － | 12 | － | － | 13 | 0 |  |
| 66 | Ⅰ-1 | 8 | 4 | 263 | 2 | 1946 | un | c.464-1G>C splicing | 66 | 66 | － | 69 | 66 | － | 0.751 | 70 | 0 |  |
|  | Ⅱ-1 |  |  | 255 | 1 | 1978 | m | c.464-1G>C splicing | 28 | 28 | － | 28 | 28 | － | 0.453 | 38 | 0 |  |
|  | Ⅱ-2 |  |  | 256 | 2 | 1973 | m | c.464-1G>C splicing | 32 | 32 | － | 36 | 36 | － | 0.385 | 43 | 0 |  |
|  | Ⅱ-3 |  |  | 259 | 2 | 1975 | m | c.464-1G>C splicing | 39 | 39 | － | 39 | 39 | － | 0.593 | 41 | 0 |  |
|  | Ⅲ-1 |  |  | 261 | 2 | 2005 | p | c.464-1G>C splicing | － | － | － | － | － | － | 0.205 | 11 | 0 |  |
|  | Ⅲ-2 |  |  | 262 | 1 | 2011 | p | c.464-1G>C splicing | － | － | － | － | － | － | 0.147 | 5 | 0 |  |
|  | Ⅲ-3 |  |  | 257 | 2 | 2004 | m | c.464-1G>C splicing | － | － | － | － | － | － | 0.291 | 12 | 0 |  |
|  | Ⅲ-4 |  |  | 258 | 2 | 2008 | m | c.464-1G>C splicing | － | － | － | － | － | － | 0.222 | 8 | 0 |  |
|  | Ⅲ-5 |  |  | 260 | 1 | 1999 | m | c.464-1G>C splicing | － | － | － | － | － | － | 0.214 | 17 | 0 |  |
| 67 | Ⅰ-1 | 1 | 0 | 293 | 2 | 1966 | n | c.464-1G>A splicing | 17 | 17 | － | － | － | － | － | 27 | 1 | CHB |
|  | Ⅱ-1 |  |  | 292 | 2 | 1991 | m | c.464-1G>A splicing | 16 | 16 | － | － | － | － | 0.276 | 25 | 0 |  |
| 68 | Ⅰ-1 | 3 | 1 | 373 | 1 | 1938 | u | c.464-2A>G splicing | 58 | － | － | 58 | － | － | － | 60 | 1 | RCC |
|  | Ⅱ-1 |  |  | 372 | 1 | 1965 | p | c.464-2A>G splicing | 51 | － | － | 51 | 51 | － | － | 51 | 0 |  |
|  | Ⅱ-2 |  |  | 374 | 2 | 1961 | p | c.464-2A>G splicing | 52 | － | － | 52 | 52 | － | － | 55 | 0 |  |
|  | Ⅲ-1 |  |  | 371 | 1 | 1989 | p | c.464-2A>G splicing | 21 | 24 | － | 21 | 21 | － | － | 27 | 0 |  |
| 69 | Ⅰ-1 | 1 | 0 | 19 | 2 | 1946 | un | c.263G>A p.Trp88Stop | 34 | 34 | － | － | － | － | 0.541 | 69 | 1 | CHB |
|  | Ⅱ-1 |  |  | 18 | 1 | 1972 | m | c.263G>A p.Trp88Stop | 34 | － | － | 34 | 38 | 39 | 0.491 | 44 | 0 |  |
| 70 | Ⅰ-1 | 2 | 1 | 25 | 1 | 1954 | p | c.280G>T p.Glu94Stop | 53 | 54 | － | 53 | 53 | － | － | 62 | 0 |  |
|  | Ⅱ-1 |  |  | 26 | 2 | 1976 | p | c.280G>T p.Glu94Stop | 35 | － | － | － | 35 | － | 0.351 | 40 | 0 |  |
|  | Ⅱ-2 |  |  | 27 | 1 | 1984 | p | c.280G>T p.Glu94Stop | 29 | － | 29 | 29 | 29 | － | － | 32 | 0 |  |
| 71 | Ⅰ-1 | 1 | 0 | 28 | 2 | 1979 | n | c.263G>A p.Trp88Stop | 28 | 30 | － | 28 | 28 | － | － | 37 | 0 |  |
|  | Ⅱ-1 |  |  | 29 | 1 | 2003 | m | c.263G>A p.Trp88Stop | － | － | － | － | － | － | 0.477 | 13 | 0 |  |
| 72 | Ⅰ-1 | 2 | 0 | 100 | 1 | 1945 | un | c.404T>A p.Leu135stop | 58 | － | － | 58 | － | － | － | 58 | 1 | RCC |
|  | Ⅱ-1 |  |  | 99 | 1 | 1972 | p | c.404T>A p.Leu135stop | 32 | 32 | － | 38 | 38 | － | 0.883 | 44 | 0 |  |
|  | Ⅲ-1 |  |  | 101 | 2 | 1999 | p | c.404T>A p.Leu135stop | 13 | 13 | － | － | － | － | － | 13 | 1 | CHB |
| 73 | Ⅰ-1 | 1 | 0 | 144 | 2 | 1972 | n | c.481C>T p.Arg161stop | 28 | 28 | － | 41 | 41 | 41 | 0.269 | 44 | 0 |  |
|  | Ⅱ-1 |  |  | 145 | 2 | 2000 | m | c.481C>T p.Arg161stop | 14 | 14 | － | － | 14 | － | -0.007 | 16 | 0 |  |
| 74 | Ⅰ-1 | 3 | 2 | 204 | 1 | 1941 | p | c.486C>A p.Cys162Stop | 49 | 49 | － | 56 | 56 | － | － | 56 | 1 | RCC |
|  | Ⅰ-2 |  |  | 205 | 1 | 1944 | p | c.486C>A p.Cys162Stop | 30 | 32 | 30 | － | － | - | － | 32 | 1 | CHB |
|  | Ⅱ-1 |  |  | 201 | 2 | 1970 | p | c.486C>A p.Cys162Stop | 28 | － | － | 44 | 28 | － | 0.833 | 46 | 0 |  |
|  | Ⅱ-2 |  |  | 203 | 1 | 1987 | p | c.486C>A p.Cys162Stop | 17 | 17 | － | － | － | － | － | 17 | 1 | CHB |
|  | Ⅲ-1 |  |  | 202 | 2 | 1997 | m | c.486C>A p.Cys162Stop | 17 | － | 17 | － | 17 | － | 0.476 | 19 | 0 |  |
| 75 | Ⅰ-1 | 2 | 2 | 325 | 2 | 1962 | un | c.337C>T p.Arg113stop | 21 | － | 21 | － | － | － | 0.727 | 54 | 0 |  |
|  | Ⅰ-2 |  |  | 326 | 2 | 1944 | un | c.337C>T p.Arg113stop | 66 | 66 | － | － | － | - | － | 72 | 0 |  |
|  | Ⅱ-1 |  |  | 323 | 1 | 1990 | m | c.337C>T p.Arg113stop | 17 | － | 17 | 26 | 26 | － | 0.596 | 26 | 0 |  |
|  | Ⅱ-2 |  |  | 324 | 2 | 1985 | m | c.337C>T p.Arg113stop | － | － | － | － | － | － | 0.392 | 31 | 0 |  |
| 76 | Ⅰ-1 | 1 | 1 | 355 | 2 | 1969 | p | c.481C>T p.Arg161stop | 40 | 40 | － | 47 | － | － | － | 47 | 0 |  |
|  | Ⅰ-2 |  |  | 356 | 1 | 1975 | p | c.481C>T p.Arg162stop | 36 | 36 | - | 36 | － | - | － | 41 | 0 |  |
|  | Ⅱ-1 |  |  | 357 | 2 | 1990 | m | c.481C>T p.Arg163stop | 14 | 14 | － | 25 | 25 | － | － | 26 | 0 |  |
| 77 | Ⅰ-1 | 1 | 1 | 384 | 2 | 1945 | u | c.481C>T p.Arg164stop | 27 | 44 | 27 | 44 | 44 | 44 | － | 44 | 1 | suicide |
|  | Ⅱ-1 |  |  | 383 | 1 | 1991 | u | c.481C>T p.Arg165stop | 21 | － | － | 21 | 21 | 21 | － | 25 | 0 |  |
|  | Ⅱ-2 |  |  | 385 | 1 | 1992 | u | c.481C>T p.Arg166stop | 22 | 22 | － | － | － | - | － | 24 | 1 | CHB |
| 78 | Ⅰ-1 | 0 | 1 | 81 | 2 | 1966 | m | c288insA Frameshift | 20 | 37 | 20 | 29 | 29 | - | 0.398 | 49 | 1 | CHB |
|  | Ⅱ-1 |  |  | 82 | 1 | 1969 | m | c289insA Frameshift | 32 | - | 32 | 43 | 43 | - | 0.466 | 47 | 0 |  |
| 79 | Ⅰ-1 | 0 | 1 | 223 | 1 | 1960 | m | c.500G>A p.Arg167Gln | 53 | － | - | 53 | － | - | 0.503 | 56 | 0 |  |
|  | Ⅱ-1 |  |  | 222 | 2 | 1963 | m | c.500G>A p.Arg167Gln | 38 | － | － | － | - | 38 | 0.416 | 53 | 0 |  |
| 80 | Ⅰ-1 | 0 | 1 | 388 | 1 | 1988 | p | c.239G>T pSer80Ile | 28 | 28 | － | － | － | - | － | 28 | 1 | CHB |
|  | Ⅱ-1 |  |  | 389 | 2 | 1994 | p | c.239G>T pSer80Ile | 22 | 22 | － | － | － | - | － | 22 | 1 | CHB |

**a**. “1” for male, “2” for female

**b**. “m” for maternal, “p” for paternal, “n” for without family history, “un” for unknown

**c**. “1” for death, “0” for alive.

**Abbreviations**: CHB, central nervous system hemangioblastoma; RA, retinal angioma; RCC, renal cell carcinoma; PCT, pancreatic cyst or pancreatic tumor; PHEO, pheochromocytoma; RTL, relative telomere length.

**Table S2 Genetic anticipation in different organs in affected parents-children pairs**

| **Organ** | **Pairs** | **Mean onset age (y)** | | **MOAD(y)** | **P**  **(paired t test)** |
| --- | --- | --- | --- | --- | --- |
|  |  | **Parent** | **Children** |  |  |
| **CHB** | 62 | 42.3±13.3 | 25.2±9.4 | 17.2 | <0.001 |
| **RCC** | 29 | 52.5±9.6 | 31.4±8.8 | 21.1 | <0.001 |
| **PCT** | 25 | 54.0±10.5 | 28.3±9.1 | 25.8 | <0.001 |
| **RA** | 10 | 35.8±12.4 | 16.5±3.1 | 19.3 | <0.001 |
| **PHEO** | 5 | 52.8±5.8 | 18.2±6.6 | 34.6 | 0.0012 |

Abbreviations: MOAD, mean of onset age difference; CHB, central nervous system hemangioblastoma; RA, retinal angioma; RCC, renal cell carcinoma; PCT, pancreatic cyst or pancreatic tumor; PHEO, pheochromocytoma.

**Table S3 Telomere length between sibling pairs**

|  | **Onset age (O>Y)** | **Onset age (O≤Y)** | **Overall** |
| --- | --- | --- | --- |
| **Age-adjusted RTL (O>Y)** | 8 | 5 | 13 |
| **Age-adjusted RTL (O≤Y)** | 3 | 3 | 6 |
| **Overall** | 11 | 8 | 19 |

Abbreviations: O, Older; Y, Younger; RTL, relative telomere length.
